# Articles on Hospital Construction

**Published:** 1919-01-25

**Authors:** 


### THE NEW ERA.

### I.—Articles on Hospital Construction.

We are proposing to publish under this heading a series of articles on the problems which confront the owners and designers of the post-war hospital. They will be practical articles, dealing in a direct way with the needs of the immediate future as affected by the conditions of the present and the experience of the past. They will treat of hospitals from the architectural rather than from the medical and surgical point of view, and will handle the questions at issue more in connection with their solution in terms of bricks and mortar than as problems of the surgical and medical professions. Naturally we draw this distinction with the greatest reservation, for the two aspects of hospital construction cannot be divorced. Hospital architecture that stands aloof from the science of hygiene is obviously of no value; equally valueless towards the advancement of hospital erection is the attitude of medicinal officials or committeemen who ignore architectural conditions. But, as long as modern medicine and modern architecture remain as they now are, studies each of which demands a good man's full energies, the combined architect-physician will remain unborn, and there will still exist an architectural side of the hospital problem.

It is, therefore, our intention in these articles to be of some service to those, whether architects, governors, practitioners, or generous donors, who are studying hospitals from the point of view which inquires, What shall we do with our old hospital? How shall we deal with our new hospital? When shall we set to work? This is a time for taking stock in every department of humanity. We are all faced with a clean slate, or at least with the knowledge that our slate can be wiped clean. No one in these days should go on in an old groove merely because it is a groove and old. Rather should we review the situation in every particular, and ask ourselves as regards every detail what good purpose does it serve and what change would serve that purpose better? We should ask again, what new expedients have come to hand? what has war taught us? what will peace bring us? what have we learned to do without? what have we found to be essential? We are all creatures of habit. Habit must be sifted and winnowed. Prejudice must be turned inside out. Old methods must be spring-cleaned, and if they drop to pieces in the cleaning they must go the way of other rubbish. The world to-day is new, and its old trappings must be discarded, except in so far as they have won through the ordeal of the change and proved themselves none the worse for their antiquity.

The new outlook in our special subject embraces a vast view of innumerable details, and we shall endeavour to select such of these as are best worthy of consideration. One article will be devoted to the bearing upon hospital construction of the multitude of temporary buildings which have been during the war erected for the accommodation of wounded and sick soldiers. Another will deal with the question of open-air treatment. Operating theatres will probably have an article to themselves, and another will be devoted to the change which may come over our hospital plans owing to an extensive development of the paying patient system. It is intended to discuss the comparative merits of the "bungalow" hospital as compared with the building several floors in height. Questions of detail, such as modern heating and ventilation, will necessarily be dealt with, as well as the broader problems of choice of site, aspect, urban *versus* rural position, and to a limited degree the selection of external and internal materials. We shall hope also, if opportunity occurs, to supply an article on the general and interesting subject of the hospital architect and his proper relation to the man of science with whose requirement he has to deal.

It is certain that we are approaching the dawn of a great activity in hospital construction. The cause of this development will very largely be identical with the impulse which is encouraging architecture in other directions also. The war suspended building operations, not, indeed, because buildings are luxuries, but because, since buildings are more or less permanent, it is sometimes a luxury to start a new building when one can "carry on" with an old one. But the armistice and the approach of peace have altered the conditions. Owners of buildings of all classes have waited under circumstances, frequently of great difficulty, for the additions, alterations, or renewals of which they were sorely in need. And, though it is true that hospitals, so far as temporary military accommodation is concerned, were exempt from the embargo, their permanent building schemes have for four years been held in abeyance. Many communities are anxious to devote their thank-offering money to the laudable purpose of the relief of suffering; many benevolent possessors of wealth are willing to help. These causes conspire to hasten the era of a new, and, let us hope, successful, outburst of hospital architecture.

